# Impact of breastfeeding and *Mycoplasma pneumoniae* co-infection on coronary artery lesions in Kawasaki disease: a retrospective cohort study

**DOI:** 10.3389/fimmu.2026.1886428

**Published:** 2026-07-27

**Authors:** YaSong Gao, FangQi Hu, XiaoChun Li, LiHua Wang, JiWang Tian, JingHui Zhang

**Affiliations:** Pediatrics Department, Anqing Municipal Hospital, Anqing, Anhui, China

**Keywords:** breastfeeding, co-infection, coronary artery lesions (CAL), Kawasaki disease, *Mycoplasma pneumoniae* infection

## Abstract

**Objective:**

This study aims to investigate the impact of different feeding patterns and *Mycoplasma pneumoniae* (MP) infection on coronary artery lesions (CAL) in children with Kawasaki disease (KD), particularly analyzing whether breastfeeding retains a protective effect against CAL in patients co-infected with MP.

**Methods:**

A retrospective cohort study was performed, recruiting 388 children diagnosed with KD between January 2020 and January 2025. The participants were categorized into three groups according to feeding status in the first 6 months of life: exclusive breastfeeding (*n* = 222), partial breastfeeding (*n* = 76), and formula feeding (*n* = 90). Of all 388 patients, 88 were MP-positive, and 56 had echocardiography-confirmed CAL. Clinical characteristics and CAL incidence were compared across feeding subgroups. Multivariate logistic regression was applied to assess interaction effects between feeding patterns and MP infection on the risk of CAL development.

**Results:**

Significant intergroup differences were detected in age, proportion of incomplete KD (iKD), and erythrocyte sedimentation rate (ESR) (*P* < 0.05). The overall CAL incidence was 14.4% (56/388), with no intergroup difference (*P* = 0.503). Platelet count was identified as an independent risk factor for CAL development (OR = 1.357, 95% CI 1.082–1.702, *P* = 0.008, per 100 × 10^9^/L increment). Multivariate logistic regression analysis did not reveal a significant interaction between feeding method and MP infection. Within the MP-positive subgroup (*n* = 88, 14 CAL events), no statistically significant difference in CAL risk was identified between the combined exclusive and partial breastfeeding and formula feeding groups.

**Conclusion:**

Among MP-positive KD children in this cohort, we detected no statistically significant inverse association between breastfeeding and CAL risk (OR = 0.906, 95% CI 0.225–3.649). The wide confidence interval reflects considerable statistical uncertainty attributable to the small number of CAL events in this subgroup; therefore, a clinically meaningful protective effect of breastfeeding cannot be ruled out.

## Introduction

Kawasaki disease (KD), first described by Dr. Kawasaki in Japan in 1967, is a febrile systemic illness characterized by vasculitis affecting small- and medium-sized arteries. Its primary complication is coronary artery lesion (CAL) (1). Although the exact etiology of KD remains incompletely elucidated, it is widely considered to arise from inflammatory cascades in genetically vulnerable hosts triggered by excessive immune responses to infectious or environmental stimuli (2). Several studies have suggested an association between KD and *Mycoplasma pneumoniae* (MP) infection (3–5). Relevant literature (3) indicates that MP infection exacerbates the progression of CAL and myocardial injury during the subacute phase of KD. Serum complement C3 and C4 concentrations serve as biomarkers of immune function and immune-mediated pathological injury. In KD patients, the peripheral blood CD4^+^ T cell counts and the CD4/CD8 ratio are elevated, while the CD8^+^ T cell numbers are reduced (6). Previous reports have documented that serum C3 concentrations in children with MP pneumonia are higher in the acute phase than the convalescent phase and remain elevated relative to those of healthy controls across both phases (7). Activation of the complement system caused by MP infection may be one of the triggering factors for MP-KD. In addition, MP can secrete superantigens that directly bind to MHC class II (MHC-II) molecules, driving the polyclonal activation of CD4+ T cells, robust cytokine release, and subsequent vascular inflammation (8, 9). Lu et al. demonstrated that IVIG effectively inhibits cytokine production, yet immune cell activation persists in KD patients with concurrent MP infection; these activated immune cells directly promote myocardial injury. This mechanism may account for the more protracted inflammation and heightened risk of cardiac sequelae observed in MP-associated KD (3). Given that concurrent MP infection markedly exacerbates long-term cardiac damage in KD patients, identifying modifiable protective interventions to reduce CAL risk in this high-risk subgroup carries important clinical implications. A Chinese case–control study reported an inverse association between exclusive breastfeeding and KD onset, implying that breastfeeding acts as a protective factor against KD (10). Furthermore, studies have shown that breastfeeding for ≥6 months significantly reduces the risk of coronary artery injury (11) and is associated with a decreased risk of KD (12, 13). Several mechanistic pathways have been proposed to account for this inverse association between breastfeeding and KD risk (10). First, breast milk contains bioactive protective factors such as immunoglobulins, nucleotides, lactoferrin, and lymphocytes, which counteract pathogenic invasion and suppress dysregulated immune responses to lower the risk of KD. Second, breastfeeding promotes infant immune maturation and confers indirect protection via reshaping intestinal microbiome composition and boosting commensal bacterial proliferation. Moreover, breastfeeding mothers typically demonstrate higher health literacy, and their infants tend to adopt healthier daily routines with lower vulnerability to infection, which further reduces KD incidence. Accordingly, breastfeeding substantially modifies the risk of immune-mediated vasculitic disorders such as KD (11). Cross-national epidemiological studies have validated breastfeeding’s protective role against KD (10–12). Nevertheless, prior systematic reviews only summarized studies verifying breastfeeding’s cardiac protective benefits among unselected KD patients (10, 11, 14); to date, no published research has evaluated whether breastfeeding modifies CAL risk specifically in KD children co-infected with MP. Accordingly, we performed this retrospective cohort study to compare CAL risk between exclusively breastfed and formula-fed children with KD complicated by MP co-infection. Partially breastfed infants were also incorporated into analyses to characterize the full spectrum of infant feeding exposures.

## Materials and methods

### Research objects and methods

We consecutively screened all children admitted to our hospital with KD between January 2020 and January 2025 and retrospectively enrolled 388 eligible participants, consisting of 232 boys and 156 girls. All participants received standardized treatment (2 g/kg IVIG + aspirin) and consistent routine clinical management. This retrospective cohort study obtained ethical approval from the Ethics Committee of Anqing Municipal Hospital; informed consent was waived given the anonymized retrospective data collection design. The study was conducted in accordance with the ethical principles of the Declaration of Helsinki.

### Inclusion and exclusion criteria

#### Diagnosis of Kawasaki disease

KD was diagnosed when patients presented with fever lasting ≥5 days plus at least four of the five core clinical manifestations (complete Kawasaki disease [cKD]), namely: (1) bilateral conjunctival congestion, (2) changes in the lips and mouth: dry and red lips, strawberry tongue, and diffuse congestion of the oropharyngeal mucosa, (3) rash, including isolated red and swollen plaques, (4) changes in the extremities: acute stage—redness and swelling of hands and feet; recovery stage—peeling of the nail beds, and (5) non-suppurative lymph node enlargement in the neck (15).

After excluding alternative febrile etiologies, patients presenting with only two or three core KD manifestations plus echocardiographically confirmed CAL were diagnosed with incomplete Kawasaki disease (iKD). Per guidelines established by the Japanese Circulation Society (16), echocardiographic CAL was defined as coronary luminal diameter ≥3 mm for children younger than 5 years, ≥4 mm for those aged 5 years or older, or a segmental diameter ≥1.5 times the adjacent coronary segments (1, 17). IVIG resistance was defined as persistent or recurrent fever ≥38.0°C occurring more than 36 h after completion of the initial IVIG infusion (15).

#### Diagnosis of *Mycoplasma* infection

MP infection was defined as concomitant positivity for both MP-IgM antibodies and MP-PCR, namely:

1. Positive PCR result from a nasopharyngeal swab collected at admission
2. Serum *Mycoplasma* IgM antibody titer ≥1.1 U/mL
3. A substantial increase in convalescent MP-IgM titers relative to acute-phase specimens served only as an auxiliary reference marker and was not required for diagnosis (most children lacked these test data in electronic medical records due to economic and medical insurance constraints).

All laboratory assays were conducted by a single trained technician following the manufacturer’s standardized protocols.

All enrolled patients underwent PCR screening for respiratory syncytial virus, adenovirus, rhinovirus, influenza A/B virus, and parainfluenza virus; only cases with negative viral test results were included.

#### Feeding pattern classification

Based on feeding practices during the first 6 months of life, data were obtained from electronic medical records or telephone follow-up with guardians. Clinical feeding data were extracted from electronic medical records for 372 of 388 participants; telephone follow-up with primary caregivers was conducted to collect feeding information for the remaining 16 children, namely:

1. Exclusive breastfeeding: Providing only breast milk to infants for the first 6 months
2. Partial breastfeeding: Received both breast milk and formula milk and/or other foods during the first 6 months
3. Formula feeding: Never received breast milk and relied entirely on formula milk

Note that for infants with KD onset before 6 months of age, feeding grouping was determined based on feeding practice from birth to disease presentation.

The exclusion criteria were as follows:

1. Concurrent infection with other respiratory pathogens (e.g., adenovirus and respiratory syncytial virus)
2. The child has concurrent genetic metabolic diseases, hematological diseases, autoimmune diseases, etc.
3. Children with missing data

### Observation indicators

We collected data by reviewing the children’s electronic medical records. The recorded information included the following:

1. Basic characteristics: age and gender
2. *Mycoplasma pneumoniae* status: antibody and PCR levels
3. Cardiac parameters: diameter of the coronary arteries measured by echocardiography (performed within 1 week of disease onset)
4. Laboratory results: including white blood cell (WBC), hemoglobin (Hb), platelet (PLT), erythrocyte sedimentation rate (ESR), C-reactive protein (CRP), alanine aminotransferase (ALT), and albumin (Alb)

### Statistical analysis

Data analysis was performed using IBM SPSS Statistics (Version 26.0). Continuous variables were expressed as mean ± standard deviation and analyzed using one-way ANOVA if they met the assumptions of normality and homogeneity of variance; otherwise, they were expressed as median (interquartile range) and analyzed using the Kruskal–Wallis *H* test (non-parametric test for *K* independent samples). Categorical variables were expressed as frequency (percentage) and analyzed using chi-square test or Fisher’s exact test. Binary logistic regression models were developed to identify independent risk factors for CAL development. We further explored the interaction effects of feeding patterns, breastfeeding duration, and MP infection on the risk of CAL. A multivariable logistic regression model was also developed within the MP-positive subgroup to assess the association between feeding patterns and CAL. Variable selection was performed by combining univariate screening with clinical judgment. Core study variables and established risk factors were forced into the regression models. Given the extremely limited number of CAL events in the MP-positive subgroup (*n* = 14), we strictly followed the events-per-variable (EPV) principle for variable entry in subgroup analyses to prevent model overfitting. Variance inflation factors (VIF) were calculated before model construction to assess multicollinearity; VIF values <2 were considered indicative of no significant collinearity. Model calibration was evaluated via the Hosmer–Lemeshow goodness-of-fit test.

## Result

A total of 388 children with Kawasaki disease were enrolled in this study, including 222 (57.2%) in the exclusive breastfeeding group, 76 (19.6%) in the partial breastfeeding group, and 90 (23.2%) in the formula feeding group. There were 232 male and 156 female patients, with a male-to-female ratio of 1.48:1. A total of 17 participants developed IVIG resistance, and CAL was detected in 56 children (14.4% of the total cohort). There were no statistically significant differences among the three groups in these two variables. With regard to age, the formula feeding group was significantly younger than the exclusive breastfeeding group (18 vs.28.5, *P* = 0.003) and had a higher proportion of iKD (14.4% vs. 5.9%, *P* = 0.013). The partial breastfeeding group exhibited the highest prevalence of iKD (19.7%) and MP co-infection (26.3%). ESR levels were significantly higher in the exclusive breastfeeding group relative to the partial breastfeeding group (*P* = 0.036), while no intergroup difference was detected between the exclusive breastfeeding and formula feeding groups (*P* = 0.241). No significant differences were found in other characteristics (*P* > 0.05) (**Table 1**).

**Table 1:** Comparison of baseline and laboratory indices in KD children by feeding method.

Variables	Exclusive breastfeeding (*N* = 222)	Partial breastfeeding (*N* = 76)	Formula feeding (*N* = 90)	*P*
Sex, male, *n* (%)	124 (55.90%)	49 (64.50%)	59 (65.60%)	0.186
Age (month)	28.50 (16–48)^b^	30.50 (16.50–48.00)^c^	18.00 (10.00–35.25)^bc^	0.002
Age stratification				
≤1 year	41 (18.50%)	11 (14.50%)^b^	29 (32.20%)^b^	0.043
1 to <5 years	157 (70.70%)	56 (73.70%)	54 (60.00%)	
≥5 years	24 (10.80%)	9 (11.80%)	7 (7.80%)	
WBC (10^9^/L)	13.56 (10.27–16.57)	12.14 (9.19–15.82)	13.06 (10.00–16.28)	0.397
Hb (g/L)	106.50 (100.00–113.00)	106.50 (101.00–115.00)	106.00 (99.00–112.25)	0.381
PLT (10^9^/L)	356.50 (282.00–450.00)	341.00 (278.25–405.00)	369.00 (285.00–474.00)	0.212
ESR (mm/h)	67.77 ± 25.30^b^	58.99 ± 25.92^b^	62.03 ± 28.36	0.023
ALT (IU/L)	23.50 (12.75–75.50)	33.50 (12.00–93.75)	30.50 (13.75–76.75)	0.423
ALB (g/L)	37.50 ± 4.17	37.88 ± 3.74	37.82 ± 4.34	0.713
CRP (mg/L)	75.61 (45.56–116.75)	70.54 (35.93–108.75)	67.55 (41.66–104.44)	0.414
Abnormal liver function, *n* (%)	68 (30.60%)	32 (42.10%)	29 (32.20%)	0.181
iKD, *n* (%)	13 (5.90%)^bc^	15 (19.70%)^c^	13 (14.40%)^b^	0.001
CAL, *n* (%)	31 (14.00%)	14 (18.40%)	11 (12.20%)	0.503
MP, *n* (%)	48 (21.60%)	20 (26.30%)	20 (22.20%)	0.696
IVIG resistance, *n* (%)	10 (4.50%)	4 (5.30%)	3 (3.30%)	0.841^a^
IVIG time (days)	6 (5–6)	6 (6–6)	6 (6–6)	0.055

WBC, white blood cell; Hb, hemoglobin; PLT, platelet; ESR, erythrocyte sedimentation rate; ALT, alanine aminotransferase; ALB, albumin; CRP, C-reactive protein; iKD, incomplete Kawasaki disease; MP, *Mycoplasma pneumoniae*; CAL, coronary artery lesion; Abnormal liver function, alanine aminotransferase > 60 IU/L; IVIG, intravenous immunoglobulin.

^a^ Fisher’s exact probability test.

^b, c^ Inter-group comparison shows P < 0.05.

Univariate logistic regression for CAL demonstrated that platelet count was the only variable significantly correlated with elevated CAL risk (*P* < 0.05); all other variables showed no statistical association. Despite the lack of statistical significance for other indicators (**Table 2**), based on clinical expertise, variables including sex, age stratification, IVIG resistance, iKD, PLT, ALT, ALB, CRP, and IVIG time were selected as potential confounding factors for subsequent multivariate logistic regression analysis.

**Table 2:** Univariate logistic regression analysis for coronary artery lesions.

Variables	OR (95% CI)	*P*
Sex		
Female	1	
Male	0.959 (0.539–1.706)	0.886
Age	0.996 (0.982–1.011)	0.633
Age stratification		
≤1 year	1	
1 to <5 years	0.984 (0.488–1.982)	0.963
≥5 years	0.821 (0.268–2.517)	0.731
Feeding practice		
Formula feeding	1	
Partial breastfeeding	1.622 (0.688–3.820)	0.269
Exclusive breastfeeding	1.166 (0.558–2.434)	0.683
iKD		
No	1	
Yes	1.795 (0.806–3.999)	0.152
IVIG resistance		
No	1	
Yes	1.888 (0.593–6.011)	0.282
IVIG time	0.730 (0.514–1.035)	0.077
*Mycoplasma* infection		
No	1	
Yes	1.162 (0.602–2.244)	0.654
Abnormal liver function		
No	1	
Yes	0.775 (0.416–1.445)	0.423
Laboratory examination		
WBC	0.975 (0.925–1.029)	0.356
Hb	1.003 (0.976–1.031)	0.816
PLT	1.313 (1.063–1.622)	0.011
ESR	0.996 (0.986–1.007)	0.510
ALT	0.998 (0.995–1.002)	0.371
ALB	1.036 (0.965–1.111)	0.328
CRP	0.997 (0.991–1.002)	0.252

WBC, white blood cell; Hb, hemoglobin; PLT, platelet; ESR, erythrocyte sedimentation rate; ALT, alanine aminotransferase; ALB, albumin; CRP, C-reactive protein; iKD, incomplete Kawasaki disease; MP, *Mycoplasma pneumoniae*; CAL, coronary artery lesion; IVIG, intravenous immunoglobulin.

After adjustment for confounders in multivariate logistic regression, no statistically significant interaction was identified between MP infection and feeding patterns, with formula feeding set as the reference category (exclusive breastfeeding * MP infection: *P* = 0.591; partial breastfeeding * MP infection: *P* = 0.119) (**Table 3**). Furthermore, after adjustment for relevant confounding factors, platelet count remained independently associated with CAL (OR = 1.357, 95% CI 1.082–1.702, *P* = 0.008, per 100 × 10^9^/L increment), corresponding to a 35.7% increase in the odds of CAL for each 100 × 10^9^/L elevation in platelet count. No other variables showed a statistically significant independent association with CAL after accounting for potential confounders.

**Table 3:** Multiplicative interaction between MP infection and feeding method.

Variables	B	SE	Wald	*P*	OR (95% CI)
*Mycoplasma* infection	0.814	0.714	1.300	0.254	2.258 (0.557–9.156)
Feeding practices					
Formula feeding	1				
Partial breastfeeding	0.955	0.538	3.146	0.076	2.598 (0.905–7.461)
Exclusive breastfeeding	0.302	0.479	0.398	0.528	1.353 (0.529–3.461)
Interaction					
Exclusive breastfeeding * *Mycoplasma* infection	-0.454	0.846	0.288	0.591	0.635 (0.121–3.335)
Partial breastfeeding * *Mycoplasma* infection	-1.710	1.096	2.432	0.119	0.181 (0.021–1.551)

Adjusted for sex, age stratification, IVIG resistance, iKD, PLT, ALT, ALB, CRP, and IVIG time.

ALT, alanine aminotransferase; ALB, albumin; CRP, C-reactive protein; PLT, platelet; iKD, incomplete Kawasaki disease; IVIG, intravenous immunoglobulin.

* represents multiplication.

In the multivariate logistic regression analysis, after adjusting for relevant confounders, compared with formula feeding, exclusive breastfeeding for <6 months (*P* = 0.248) and ≥6 months (*P* = 0.689), respectively, were not significantly associated with the risk of CAL (**Table 4**).

**Table 4:** Multivariate logistic regression analysis of breastfeeding duration and its interaction with *Mycoplasma* infection on CAL.

Variables	β	SE	Wald	*P*	OR (95% CI)
Mycoplasma infection	0.291	0.672	0.187	0.665	1.338 (0.358–4.992)
Feeding duration					
Exclusive breastfeeding (<6 months)	0.541	0.468	1.334	0.248	1.718 (0.686–4.303)
Exclusive breastfeeding (≥6 months)	0.181	0.453	0.160	0.689	1.199 (0.494–2.912)
Interaction					
Feeding duration * *Mycoplasma* infection	-0.080	0.434	0.034	0.853	0.923 (0.394–2.159)

Adjusted for sex, age stratification, IVIG resistance, iKD, PLT, ALT, ALB, CRP, and IVIG time.

ALT, alanine aminotransferase; ALB, albumin; CRP, C-reactive protein; PLT, platelet; iKD, incomplete Kawasaki disease; IVIG, intravenous immunoglobulin.

* represents multiplication.

Subgroup analyses restricted to MP-positive children included 14 cases with CAL. Compared with formula feeding, exclusive breastfeeding showed no significant modification of CAL risk (*P* = 0.890) (**Table 5**).

**Table 5:** Comparison of breastfeeding vs. formula feeding in children with MP infection.

Variables	CAL, *n* (%)	*β*	SE	Wald	*P*	OR (95% CI)
Feeding practices						
Formula feeding	4 (28.6%)	1				
Partial breastfeeding	2 (14.3%)	-0.901	0.967	0.868	0.352	0.406 (0.061–2.702)
Exclusive breastfeeding	8 (57.1%)	-0.098	0.711	0.019	0.890	0.906 (0.225–3.649)

Adjusted for age stratification and PLT.

CAL, coronary artery lesion; PLT, platelet.

## Discussion

In this study, we conducted a retrospective analysis to investigate whether breastfeeding retains a protective effect against coronary artery lesions specifically in children with KD co-infected with MP. First, no statistically significant interaction was detected between MP infection and feeding method in the overall cohort. Additionally, exclusive breastfeeding for <6 months and ≥6 months, respectively, were not significantly associated with CAL risk. Second, within the specific subgroup of MP-positive patients, exclusive breastfeeding did not significantly reduce the risk of CAL compared to formula feeding. Collectively, these findings did not reveal a statistically significant inverse association between breastfeeding and CAL risk in our MP-positive KD patients.

Many studies have reported that infectious pathogens play an important role in KD, and MP is one of the most frequently reported microorganisms causing KD (17). Several preclinical studies indicate that mitophagy participates in KD pathogenesis (18). In murine models of MP-triggered KD, PINK1/Parkin-dependent mitophagy was markedly activated; blocking this signaling cascade alleviated systemic disease phenotypes (5).

Currently, breastfeeding has become the preferred method for infants to obtain nutrition. In this study, 57% of the enrolled children were breastfed. In terms of age at KD onset, the partial breastfeeding group had the highest median age, whereas the formula feeding group presented with the lowest. Laboratory analysis revealed that the ESR was highest in the exclusive breastfeeding group, showing a statistically significant difference compared to the partial breastfeeding group. Notably, white blood cell, hemoglobin, and platelet counts did not differ significantly across the three groups, suggesting that feeding patterns exert minimal influence on routine hematological markers in KD patients, which are consistent with the results of a prior work by Wang et al. (19) In their retrospective cohort study of 568 children with KD, the authors concluded that breastfeeding conferred no significant protective effect against CAL. Neither study yielded statistical evidence supporting a protective role of breastfeeding in reducing CAL risk. Nevertheless, notable discrepancies existed between the two investigations. Wang’s study included 149 CAL cases and did not specifically address the subgroup of patients with concurrent MP infection. In contrast, our study recorded only 56 CAL events overall, and we performed exploratory analyses specifically for the MP-positive subgroup. No breastfeeding-related protective association was detected within this subgroup; nevertheless, only 14 CAL outcomes were recorded, which drastically reduced the statistical power for stratified analyses. Both cohorts enrolled Chinese children with comparable geographic and ethnic characteristics, which improves the consistency of pooled evidence from separate clinical centers. Overall, data from both studies fail to provide robust evidence confirming a consistent protective benefit of breastfeeding against CAL in KD children. However, due to the retrospective design of both cohorts and insufficient statistical power in subgroup stratification, large-scale multicenter prospective studies are required to verify these observations.

CRP, commonly elevated in both infection and inflammation, is a well-recognized biomarker in the diagnosis of suspected iKD (15). In this study, the CRP levels were lower in the iKD group than in the cKD group (53.59 [17.03–109.70] vs. 73.68 [45.58–111.60], *P* = 0.019), which aligns with previous studies (20). However, the distribution of KD subtypes (iKD vs. cKD) differed significantly among the three feeding groups. The partial breastfeeding group had the highest proportion of iKD (19.7%), while the exclusive breastfeeding group had the lowest (5.9%), indicating a significantly higher iKD prevalence in the partial breastfeeding group compared to the exclusive breastfeeding group. In clinical practice, iKD often presents with atypical symptoms, which may lead to misdiagnosis or delayed treatment, thereby hindering early standardized intervention. It should be emphasized that the comparison of KD subtype distribution in this study was a non−prespecified secondary exploratory analysis rather than the primary study outcome. Due to the inherent limitations of this retrospective observational study, we can only confirm a statistical association between feeding patterns and KD subtype phenotypes; no causal relationship can be inferred from the current data. The occurrence and diagnosis of iKD are also influenced by multiple confounding factors, including clinicians’ diagnostic experience and the early symptomatic presentation of the disease, which were not fully adjusted for in this study. Therefore, the observed intergroup difference should be regarded solely as a hypothesis−generating reference for future investigations and cannot be interpreted as evidence that breastfeeding reduces the risk of atypical KD presentation. Further validation through large-scale prospective studies is warranted to clarify this association. In contrast, clinical outcome measures including CAL and IVIG resistance showed no significant differences among the three feeding groups.

Multivariable analysis revealed that platelet count was the only independent predictor of CAL (OR = 1.357, 95% CI: 1.082–1.702, *P* = 0.008, per 100 × 10^9^/L increment). This observation suggests that thrombocytosis is not merely a secondary epiphenomenon of systemic inflammation in KD but may serve as a marker of a more severe inflammatory pathology associated with CAL development. The literature indicates that CAL is closely linked to inflammation, immune dysregulation, and endothelial dysfunction, in which platelets play a crucial role through complex molecular networks (21, 22). As critical immune effector cells in KD, platelets amplify inflammatory signaling through crosstalk with leukocytes and endothelial cells (e.g., platelet–monocyte aggregates exacerbate vascular inflammation) (23, 24) and elevate thrombotic risk via TSLP-mediated mitophagy (25). Furthermore, downregulation of platelet-derived miR-223 relieves the suppression of abnormal dedifferentiation in vascular smooth muscle cells (26). Thus, platelet count serves as a reliable surrogate marker for these underlying pathological changes, explaining its role as an independent predictor of CAL. Accordingly, clinicians should closely monitor dynamic changes in platelet counts in KD patients, intensify echocardiographic follow-up and surveillance, and promptly initiate interventions to reduce the risk of coronary artery complications.

While platelet count remained a significant independent risk factor in multivariate regression, the interaction terms between our primary exposures (feeding patterns and MP infection as well as breastfeeding duration and MP infection) did not attain statistical significance (*P* > 0.05). Stratified analyses limited to MP-positive participants further confirmed the absence of a statistically significant link between feeding patterns and CAL occurrence. Importantly, these non-significant results were derived from a small subgroup with merely 14 CAL endpoints, generating broad confidence intervals (OR = 0.906, 95% CI 0.225–3.649) and limited statistical power to identify clinically relevant associations. Accordingly, these non-significant outcomes cannot be interpreted as proof that breastfeeding lacks protective effects; they instead represent inconclusive findings caused by inadequate statistical power. Baseline data showed that the proportions of iKD and MP infection in the partial breastfeeding group were 19.7% and 26.3%, respectively, both higher than those in the exclusive breastfeeding group (5.9% and 21.6%). However, after adjusting for multiple confounding factors, these intergroup differences were not statistically significant. Therefore, the current data were insufficient to confirm an independent association between partial breastfeeding and atypical clinical manifestations or MP infection. This finding was not a primary outcome of the present study but, rather, an exploratory observation; further large-scale studies are needed to validate these trends.

Admittedly, this single-center retrospective cohort study has several inherent limitations that must be acknowledged. First, feeding-related information was collected retrospectively via electronic medical records and telephone follow-ups with guardians. For a small subset of patients, a long interval existed between the infant feeding period and data collection, which predisposed guardians to recall bias when reporting early feeding details. This bias was more pronounced among older children, making it difficult to accurately determine breastfeeding duration. Second, convalescent serum titers were not tested to confirm MP infection, so residual confounding caused by persistent IgM positivity could not be fully ruled out. Third, this was a single-center retrospective design with inherent selection bias, limiting the generalizability of our conclusions. Fourth, the overall number of CAL cases was small; only 14 CAL events were identified in the MP-positive subgroup, leading to low statistical power and a risk of type II error, which compromised the reliability of interaction analyses. Fifth, although the regression models were adjusted for multiple well-established risk factors, several potential confounders including duration of fever before IVIG treatment, diagnostic delay, socioeconomic status, and maternal factors could not be incorporated, resulting in residual confounding. Sixth, CAL was defined based on absolute coronary artery diameters rather than body-surface-area-adjusted *Z*-scores as recommended by the AHA (American Heart Association). Combined with significant intergroup differences in age distribution, this approach may have introduced CAL misclassification bias. Thus, multicenter, large-sample prospective studies with standardized coronary *Z*-score measurements and comprehensive collection of confounders are warranted to further validate the findings of the present study.

## Conclusion

In summary, we detected no statistically significant protective association between breastfeeding and CAL among MP-co-infected KD children within our cohort (OR = 0.906, 95% CI 0.225–3.649). Nevertheless, broad confidence intervals reflect considerable statistical uncertainty, and *post-hoc* power analysis revealed limited power to detect clinically meaningful protective effects. Therefore, these results should not be interpreted as evidence of no protective effect but, rather, as inconclusive findings due to inadequate sample size and low statistical power. A clinically relevant protective effect of breastfeeding in MP-co-infected KD patients cannot be excluded, and large-scale prospective multicenter studies are required to resolve this research question.

## Data Availability

The raw data supporting the conclusions of this article will be made available by the authors, without undue reservation.

## References

[1] Harahsheh AS Mohsin SM Tremoulet A McCrindle BW Singh S Webb K . Importance of fostering international collaboration for optimal outcomes of Kawasaki disease worldwide: A science advisory from the American Heart Association. J Am Heart Assoc. (2026) 15:e050184. doi: 10.1161/jaha.126.15.050184 42145156 PMC13477275

[2] Shulman ST Rowley AH . Kawasaki disease: Insights into pathogenesis and approaches to treatment. Nat Rev Rheumatol. (2015) 11:475–82. doi: 10.1038/nrrheum.2015.54 25907703

[3] Lu G Li X Tang J Jin Y Wang Y Zhou K . Mycoplasma infection aggravates cardiac involvements in Kawasaki diseases: A retrospective study. Front Immunol. (2023) 14:1310134. doi: 10.3389/fimmu.2023.1310134 38304251 PMC10832023

[4] Plebani A Meini A Cattalini M Lougaris V Bugatti A Caccuri F . Mycoplasma infection may complicate the clinical course of SARS-Co-V-2 associated Kawasaki-like disease in children. Clin Immunol. (2020) 221:108613. doi: 10.1016/j.clim.2020.108613 33069853 PMC7561565

[5] Wang C Zhang H Zhang J Hong Z Miao C Wang T . Mycoplasma pneumoniae-induced Kawasaki disease via PINK1/Parkin-mediated mitophagy. Exp Cell Res. (2024) 441:114182. doi: 10.1016/j.yexcr.2024.114182 39094903

[6] Ding Y Li G Xiong LJ Yin W Liu J Liu F . Profiles of responses of immunological factors to different subtypes of Kawasaki disease. BMC Musculoskelet Disord. (2015) 16:315. doi: 10.1186/s12891-015-0744-6 26497060 PMC4619387

[7] Mi YM Qi Q Zhang L Wang XF Chen ZM Hua CZ . Assessment of serum sialic acid correlated with C3 in children with Mycoplasma pneumoniae pneumonia. J Clin Lab Anal. (2020) 34:e23078. doi: 10.1002/jcla.23078 31907994 PMC7083476

[8] Shio MT Hassan GS Shah WA Nadiri A El Fakhry Y Li H . Coexpression of TLR2 or TLR4 with HLA-DR potentiates the superantigenic activities of Mycoplasma arthritidis-derived mitogen. J Immunol. (2014) 192:2543–50. doi: 10.4049/jimmunol.1300591 24493819

[9] Lan Y Li S Yang D Zhou J Wang Y Wang J . Clinical characteristics of Kawasaki disease complicated with Mycoplasma pneumoniae pneumonia: A retrospective study. Med (Baltimore). (2020) 99:e19987. doi: 10.1097/md.0000000000019987 32384451 PMC7220055

[10] Wang S Xiang D Fang C Yao B . Association between breastfeeding and Kawasaki disease: A case-control study. Eur J Pediatr. (2020) 179:447–53. doi: 10.1007/s00431-019-03529-y 31797082

[11] Yorifuji T Tsukahara H Doi H . Breastfeeding and risk of Kawasaki disease: A nationwide longitudinal survey in Japan. Pediatrics. (2016) 137:e050184. doi: 10.1542/peds.2015-3919 27244853

[12] Meyer K Volkmann A Hufnagel M Schachinger E Klau S Horstmann J . Breastfeeding and vitamin D supplementation reduce the risk of Kawasaki disease in a German population-based case-control study. BMC Pediatr. (2019) 19:66. doi: 10.1186/s12887-019-1438-2 30808315 PMC6390341

[13] Yang WJ Lu WH Hsiao YY Hsu TW Chiou YH . Protective effect of breastfeeding on Kawasaki disease: A systemic review and meta-analysis. Pediatr Neonatol. (2024) 65:427–34. doi: 10.1016/j.pedneo.2024.03.001 38627111

[14] Takeuchi A Namba T Matsumoto N Tamai K Nakamura K Nakamura M . Preterm birth and Kawasaki disease: A nationwide Japanese population-based study. Pediatr Res. (2022) 92:557–62. doi: 10.1038/s41390-021-01780-4 34625654

[15] McCrindle BW Rowley AH Newburger JW Burns JC Bolger AF Gewitz M . Diagnosis, treatment, and long-term management of Kawasaki disease: A scientific statement for health professionals from the American Heart Association. Circulation. (2017) 135:e927–99. doi: 10.1161/cir.0000000000000484 28356445

[16] Guidelines for diagnosis and management of cardiovascular sequelae in Kawasaki disease (JCS 2013). Digest version. Circ J. (2014) 78:2521–62. doi: 10.1111/j.1442-200x.2005.02149.x 25241888

[17] Tang Y Yan W Sun L Huang J Qian W Hou M . Kawasaki disease associated with Mycoplasma pneumoniae. Ital J Pediatr. (2016) 42:83. doi: 10.1186/s13052-016-0292-1 27609267 PMC5016862

[18] Marek-Iannucci S Ozdemir AB Moreira D Gomez AC Lane M Porritt RA . Autophagy-mitophagy induction attenuates cardiovascular inflammation in a murine model of Kawasaki disease vasculitis. JCI Insight. (2021) 6(18):e151981. doi: 10.1172/jci.insight.151981 34403365 PMC8492304

[19] Wang H Tang Y Yan W Xu Q Li X Qian W . Breastfeeding has no protective effects on the development of coronary artery lesions in Kawasaki disease: A retrospective cohort study. BMC Pediatr. (2022) 22:353. doi: 10.1186/s12887-022-03422-y 35725463 PMC9208131

[20] Kil HR Yu JW Lee SC Rhim JW Lee KY . Changes in clinical and laboratory features of Kawasaki disease noted over time in Daejeon, Korea. Pediatr Rheumatol Online J. (2017) 15:60. doi: 10.1186/s12969-017-0192-y 28784161 PMC5545846

[21] Huang T Peng Q Zhang Y Zhu Z Fan X . The systemic immune-inflammation index (SII) and coronary artery lesions in Kawasaki disease. Clin Exp Med. (2024) 24:4. doi: 10.21203/rs.3.rs-3449129/v1 38231301 PMC10794328

[22] Yang M Pei Q Zhang J Weng H Jing F Yi Q . Association between adropin and coronary artery lesions in children with Kawasaki disease. Eur J Pediatr. (2021) 180:2253–9. doi: 10.1007/s00431-021-03977-5 33712900

[23] Noval Rivas M Kocatürk B Franklin BS Arditi M . Platelets in Kawasaki disease: Mediators of vascular inflammation. Nat Rev Rheumatol. (2024) 20:459–72. doi: 10.1038/s41584-024-01119-3 38886559

[24] Zhang Y Jia C Guo M Chen Q Wen Y Wang T . Platelet-monocyte aggregate instigates inflammation and vasculopathy in Kawasaki disease. Adv Sci (Weinh). (2025) 12:e2406282. doi: 10.1002/advs.202406282 39665236 PMC11792051

[25] Fu L MacKeigan DT Gong Q Che D Xu Y Pi L . Thymic stromal lymphopoietin induces platelet mitophagy and promotes thrombosis in Kawasaki disease. Br J Haematol. (2023) 200:776–91. doi: 10.1111/bjh.18531 36341698

[26] Zhang Y Wang Y Zhang L Xia L Zheng M Zeng Z . Reduced platelet miR-223 induction in Kawasaki disease leads to severe coronary artery pathology through a miR-223/PDGFRβ vascular smooth muscle cell axis. Circ Res. (2020) 127:855–73. doi: 10.1161/circresaha.120.316951 32597702 PMC7486265

